# Polypill Therapy for Cardiovascular Disease Prevention and Combination Medication Therapy for Hypertension Management

**DOI:** 10.3390/jcm12237226

**Published:** 2023-11-22

**Authors:** Keisuke Narita, Satoshi Hoshide, Kazuomi Kario

**Affiliations:** Division of Cardiovascular Medicine, Department of Internal Medicine, Jichi Medical University School of Medicine, Shimotsuke 329-0498, Japan

**Keywords:** polypill strategy, combination medication therapy, cardiovascular prevention, blood pressure, hypertension

## Abstract

Although various guidelines for cardiovascular disease prevention have been established, the optimal drug therapy is often not implemented due to poor medication adherence and the clinical inertia of healthcare practitioners. Polypill strategies are one solution to this problem. Previous studies have established the usefulness of polypills, i.e., combination tablets including three or more medications, for the prevention of cardiovascular disease. For this purpose, the polypills generally contain an antiplatelet medication, an antihypertensive medication, and a statin. For the specific management of hypertension, combination therapy including more than two classes of antihypertensive medications is recommended by most international guidelines. Combination tablets including two classes of antihypertensive medications, such as renin-angiotensin system (RAS) inhibitors (angiotensin-converting enzyme inhibitors [ACEIs] and angiotensin receptor blockers [ARBs]) and Ca-channel blockers or thiazide diuretics, have been reported to be useful for cardiovascular disease prevention and lowering blood pressure (BP) levels. The use of RAS inhibitors is recommended for a wide range of complications, including diabetes, chronic heart failure, and chronic kidney disease. The combination of an RAS inhibitor and diuretic or Ca-channel blocker is thus recommended for the management of hypertension. Finally, we expect that novel medications such as angiotensin receptor neprilysin inhibitors (ARNIs) and sodium glucose cotransporter 2 inhibitors (SGLT2i), which have a more diverse range of effects in hypertension, heart failure, or diabetes, may be a solution to the problem of polypharmacy. Evidence is accumulating on the benefits of polypill strategies in cardiovascular disease prevention. Combination tablets are also effective for the treatment of hypertension.

## 1. Introduction

Cardiovascular disease (CVD) is one of the most common causes of disability and death worldwide. Many guidelines for the prevention of CVD have been established in Asia, Europe, and the United States. However, the optimal drug therapy recommended in the guidelines is not being implemented in all patients due to poor adherence to treatment medications and a lack of prescribing due to clinical inertia in healthcare practitioners [1,2,3,4].

One major obstacle to the implementation of optimal therapy is poor medication compliance, and one major reason for poor medication compliance is the number of pills in the therapeutic regimen. In the case of CVD, complications that place patients at high risk include hypertension, diabetes, and dyslipidemia, and these diseases often co-occur and require multidrug therapy. Combination pills, which combine two or more drugs into a single pill, are a possible solution to this problem [3,5,6].

In the field of hypertension management, a substantial population of untreated hypertensive patients exists not only in Japan but also in other developed countries, including the United States and Europe. Moreover, the persistently low success rate in reaching treatment objectives poses a significant challenge in hypertension management. The Japanese Hypertension Guidelines refer to this issue as the “hypertension paradox” and view it as a concern, despite the relatively straightforward diagnostic methods available for hypertension, such as blood pressure measurement, and the availability of numerous highly effective antihypertensive medications [2]. Poor medication adherence and healthcare provider inertia are frequently cited as reasons for the suboptimal achievement of target BP levels in the treatment of hypertension. Utilizing combination tablets for hypertension therapy could potentially offer a viable solution to address these challenges.

In this review, the usefulness of polypills in CVD prevention and medication adherence is presented. We also present the evidence and our opinion on the usefulness of the combination therapy of antihypertensive medications in the management of hypertension.

## 2. Polypill Strategies for the Cardiovascular Disease Prevention

Several previous studies have established the usefulness of polypill treatment for the prevention of CVD [5,6,7,8,9,10,11]. With regard to the benefit of combination therapy with aspirin, statins and antihypertensives, polypills have been shown to be more effective than usual care. [12,13]. Table 1 shows a summary of the major clinical trials designed to assess the effect of polypill strategies for medication adherence and the prevention of CVD events. In terms of the effects of polypills on the primary and secondary prevention of CVD, Huffman et al. summarized 13 polypill trials and concluded that polypill therapy could be one of the most scalable strategies to reduce the risk of premature cardiac death, resulting in a 25% reduction in premature cardiac death by 2025 through an improvement in medication adherence and access [8]. The recent PolyIran study, a pragmatic, cluster-randomized trial conducted as part of the Golestan Cohort Study, found that polypills that included aspirin, atorvastatin, hydrochlorothiazide, and either enalapril or valsartan were useful for achieving the high adherence of medications and noted a reduction in the risk of major CVD events compared to those of patients receiving minimal care (HR 0.66, 95% CI 0.55–0.80) [9]. Merat et al. conducted a randomized controlled trial as a sub-study of the PolyIran-Liver trial in order to assess the effects of polypills including an angiotensin receptor blocker (ARB), a thiazide diuretic, a statin, and aspirin in patients with non-alcoholic steatohepatitis, and they demonstrated that the polypill is more useful for the prevention of CVD events compared to multiple tablets administered separately [14].

Previous studies have suggested the benefit of including aspirin in polypill therapy. Yusuf et al. examined the effect of adding aspirin to polypill therapy on the prevention of cardiovascular events in patients without cardiovascular disease by using a two-by-two-by-two factorial design that included a double placebo, aspirin, a polypill (simvastatin, atenolol, hydrochlorothiazide, and ramipril), and a polypill-plus-aspirin. In participants without cardiovascular disease and at intermediate risk for cardiovascular disease, the polypill-plus-aspirin therapy reduced the incidence of cardiovascular events compared with the double placebo group [15]. Moreover, Joseph et al. conducted an individual patient-level meta-analysis of three large, controlled trials (TIPS-3, HOPE-3, and PolyIran: n = 18,162). With a median follow-up of 5 years, a primary outcome benefit, including cardiovascular death, myocardial infarction, and stroke, of the polypill strategy was observed (HR: 0.62). Sub-analysis with and without aspirin showed a greater risk reduction with the aspirin-containing strategy [16]. From these findings, the polypill strategy is useful for the prevention of CVD incidence [17,18,19]. Secondarily, these combination drugs used for the secondary prevention of CVD also include beta-blockers such as atenolol, metoprolol, and others [15,20]. Although beta-blockers are useful in the secondary prevention of coronary artery disease and the treatment of heart failure and have cardiovascular protective effects, adverse effects such as bradycardia and syncope should be considered [21].

### 2.1. Polypill Therapy for Secondary Prevention of Cardiovascular Disease

To assess the usefulness of polypills for the secondary prevention of CVD, the randomized controlled Secondary Prevention of Cardiovascular Disease in Elderly (SECURE) trial was conducted in 2499 patients in Europe. This study demonstrated that a polypill including an angiotensin-converting enzyme inhibitor (ACEI), a statin, and aspirin significantly improved CVD outcomes compared to the usual care (HR 0.76, 95% CI 0.60–0.96 for composite CVD events including CVD death) [10]. In terms of the secondary prevention of CVD, polypill treatment is acceptable for use in clinical practice. In addition, studies of polypill treatment strategies for acute coronary syndromes have recently been underway. These studies have employed polypill therapy, i.e., combination tablets, containing antiplatelet agents and statins. It is anticipated that evidence will continue to accumulate regarding the utility of polypills in the treatment of acute coronary syndromes.

### 2.2. Polypill Therapy and Medication Adherence

Many previous studies have established evidence regarding the usefulness of the polypill strategy for the improvement of medication adherence [9,22,23,24,25]. In the Use of a Multidrug Pill in Reducing Cardiovascular Events (UMPIRE) trial, a randomized control trial designed to assess the effectiveness of fixed-dose combinations (polypills), combination drug treatment was shown to achieve greater reductions in BP, cholesterol, and platelet control compared to multiple tablets administered separately [22]. In addition, many healthcare providers consider the polypill beneficial in terms of improving patient adherence and reducing drug costs [26,27,28,29]. A polypill strategy is also important for preventing CVD events in low-to-medium-income countries and communities [30]. Polypill treatments were reported to realize greater reductions in systolic blood pressure (BP) and LDL cholesterol levels than the usual care in a socioeconomically vulnerable minority population in the United States. Muñoz, et al. conducted an interventional trial to assess the efficacy of a polypill (atorvastatin, amlodipine, losartan, and hydrochlorothiazide) treatment group compared to a usual treatment group in 303 patients in low-income regions [31]. From these results of previous studies, polypill therapy is useful in reducing the costs of treatment and preventing primary and secondary CVD events in lower- or middle-income regions.

## 3. Combination Therapy of Antihypertensive Medications in the Management of Hypertension

Compared to monotherapy, combination tablets have gained rapid popularity in recent years due to their superior antihypertensive effects, improved patient adherence, and health economic benefits. Furthermore, not only are combination tablets of antihypertensive drugs utilized but combination formulations with diabetes drugs and other types of medications are also employed in clinical practice. Hypertensive patients often present with additional cardiovascular risk factors, including a history of cardiovascular disease, lipid abnormalities, diabetes, and obesity. In these cases, the number and variety of oral medications can be substantial, resulting in challenges related to reduced patient adherence and increased healthcare costs. Combination tablets of antihypertensive drugs are expected to offer a solution to these issues. Most international guidelines for the management of hypertension recommend a combination therapy of renin-angiotensin system (RAS) inhibitors (ACEIs and ARBs), Ca-channel blockers, and diuretics [2,32,33]. The World Health Organization (WHO) guideline for the pharmacological treatment of hypertension recommends combination therapy chosen from the above-mentioned three classes of antihypertensive medications, such as thiazide diuretics, ACEIs/ARBs, and Ca-channel blockers [34]. In this WHO guideline, it is mentioned that combination medication therapy may be especially valuable when the baseline BP is more than SBP 20 mmHg higher than the target BP level, and single-pill combination therapy improves medication-taking adherence and persistence and BP control [34].

Many interventional trials have provided evidence of the usefulness of combination therapy with antihypertensive medications for lowering BP levels and preventing CVD events. The combination of RAS inhibitors (ACEIs or ARBs) and diuretics has been shown to be useful for preventing CVD events in randomized control trials [35]. In the ADVANCE trial, Patel, et al. demonstrated that a fixed combination of ACEI (perindopril) and thiazide diuretic (indapamide) treatment reduced major cardiovascular events by 9% compared to placebo (HR 0.91, 95% CI 0.83–1.00, *p* = 0.04) [36]. In addition, the combination of RAS inhibitors and Ca-channel blockers was also reported to be useful for the prevention of CVD [37,38,39]. Based on the findings from these studies, RAS inhibitors (ACEIs or ARBs) and diuretics or Ca-channel blockers are a reasonable choice for a combination of antihypertensive medications. European guidelines also recommend this combination [33]. RAS inhibitors are recommended for use in a wide range of diseases, such as diabetes, left ventricular hypertrophy, chronic heart failure, and chronic kidney disease. There are some advantages to using RAS inhibitors with diuretics, such as a decreased risk of diuretic-induced hypokalemia. However, side effects and patient tolerability should be noted.

Recently, a polypill strategy using a quad-pill including an RAS inhibitor, a Ca-channel blocker, a diuretic, and a beta-blocker has been reported. Chow et al. conducted an interventional trial to assess the effect of a quad-pill containing irbesartan, amlodipine, indapamide, and bisoprolol for lowering BP and observed significantly better BP control in the quad-pill group compared to the monotherapy group (relative risk 1.30, 95% CI 1.15–1.47), with no difference in adverse events between the two groups [20].

The WHO guidelines emphasize the significance of treating hypertension in developing countries and non-urban areas characterized by remote and impoverished economic conditions [40]. The polypill treatment strategy has proven to be effective in cardiovascular prevention within regions marked by economic hardship, particularly in terms of improving drug adherence. Similarly, a two-drug combination for hypertension treatment may offer benefits in regions with limited economic resources and low health literacy.

## 4. Combination Therapy of Renin-Angiotensin System (RAS) Inhibitors and Ca-Channel Blockers or Thiazide Diuretics

**Figure 1:**
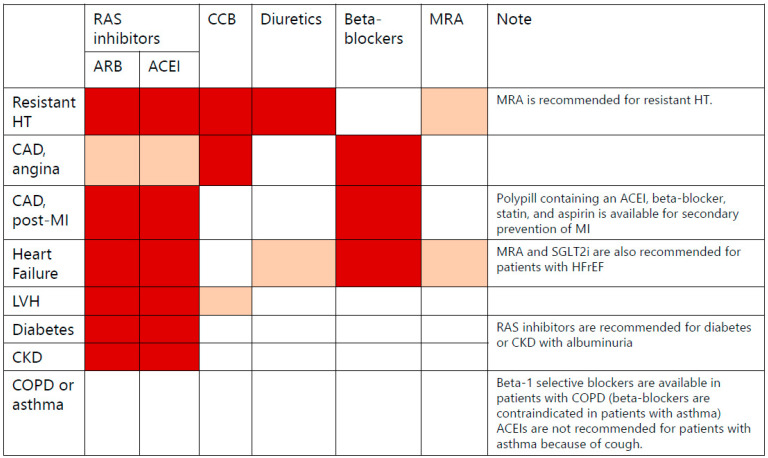
Strategy for combination therapy of antihypertensive medications. The international guidelines recommend the combination: renin angiotensin system (RAS) inhibitors and Ca-channel blockers or diuretics. RAS inhibitors are recommended for use in a wide range of conditions, such as diabetes, left ventricular hypertrophy, chronic heart failure, and chronic kidney disease. Red colour, highly recommended; Beige colour, recommended.

RAS inhibitors (ACEIs or ARBs), along with diuretics or calcium channel blockers, constitute a rational choice for a combination of antihypertensive medications. European guidelines also advocate for this particular combination [33]. RAS inhibitors are recommended for application in a broad spectrum of conditions including diabetes, left ventricular hypertrophy, chronic heart failure, and chronic kidney disease, further emphasizing their versatility in the management of hypertension. Figure 1 shows recommendation of antihypertensive medications for each comorbidity. Subsequently, many studies assessing the effectiveness of combination therapy of antihypertensive medications have not incorporated out-of-office blood pressure measurements, such as ambulatory BP monitoring (ABPM) and home BP monitoring, in their evaluation of BP control. These out-of-office BP measurements including ABPM and home BP monitoring have been recognized as more valuable than office BP measurements for stratifying cardiovascular risk and are recommended in clinical guidelines [2,32,33]. Therefore, the evaluation of combination therapy with antihypertensive drugs should also include an assessment of their antihypertensive efficacy using out-of-office BP measurements.

**Figure 2:**
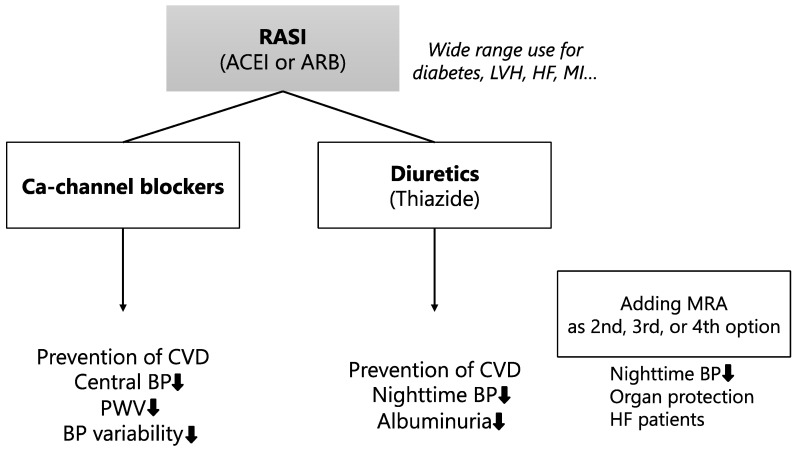
Strategy for combination therapy of antihypertensive medications. The international guidelines recommend the combination: renin angiotensin system (RAS) inhibitors and Ca-channel blockers or diuretics. RAS inhibitors are recommended for use in a wide range of conditions, such as diabetes, left ventricular hypertrophy, chronic heart failure, and chronic kidney disease.

The usefulness of combination tablets including RAS inhibitors and diuretics or Ca-channel blockers has been reported (Figure 2) [33,41,42]. It has been reported that the standard-dose combination of ARB and Ca-channel blockers is superior to high-dose Ca-channel blocker monotherapy for lowering BP and reducing adverse events in hypertensive patients [43]. Moreover, Filipova et al. have reported that the combination tablet of ARB and hydrochlorothiazide showed a greater reduction in BP compared with ARB in a meta-analysis [44]. In the J-CORE study, it has been reported that a combination tablet containing the ARB olmesartan and the Ca-channel blocker azelnidipine has achieved a greater reduction in central BP and measured pulse wave velocity (PWV) compared to olmesartan and hydrochlorothiazide tablets. In addition, the same study has reported that the olmesartan and azelnidipine combination tablet has also achieved a greater reduction in home BP variability compared to the olmesartan and hydrochlorothiazide combination tablet [45]. On the other hand, olmesartan and hydrochlorothiazide tablets reduced nighttime BP and albuminuria more strongly than the olmesartan and azelnidipine combination tablet [46]. Nighttime BP is related to hypertensive target organ damage such as renal dysfunction and is an important treatment target in the management of hypertension since it is a better predictor of CVD compared to daytime BP [47]. In addition, it has been reported that for patients with poorly controlled BP on ARBs, the addition of mineralocorticoid receptor antagonist (MRA) (eplerenone) significantly reduced nighttime BP levels [48]. Previous studies have suggested that the combination therapy of RAS inhibitors (ACEIs or ARBs) and Ca-channel blockers or diuretics may be useful for lowering BP levels and conferring protection against hypertensive organ damage. A recent meta-analysis has reported that combination tablets of ARBs and Ca-channel blockers show superior cardiovascular disease prevention compared with combination tablets of ARBs and diuretics [49]. However, more evidence needs to be accumulated through new intervention studies. Internists should use a combination tablet of an ARB and Ca-channel blockers or a combination tablet of an ARB and diuretics, depending on the individual pathological condition of the hypertensive patient. Combination therapy with RAS inhibitors as the main axis and CCB or diuretics added as recommended in the guidelines is a reasonable choice for hypertension management [42].

## 5. Novel Medications with Diverse Effects

The goal of polypill therapy is to improve medication adherence and quality of treatment by reducing the number of tablets. In recent years, novel medications have become available that exert more effects via a single tablet compared to conventional medications for the treatment of hypertension, heart failure, or diabetes. Angiotensin receptor neprilysin inhibitor (ARNI) and sodium glucose cotransporter 2 inhibitor (SGLT2i) are two promising examples [50,51,52,53]. These drugs have the potential to combine the effects of two drugs into a single drug and may be a solution to the problem of polypharmacy in recent clinical practice.

### 5.1. Angiotensin Receptor Neprilysin Inhibitors (ARNI)

**Figure 3:**
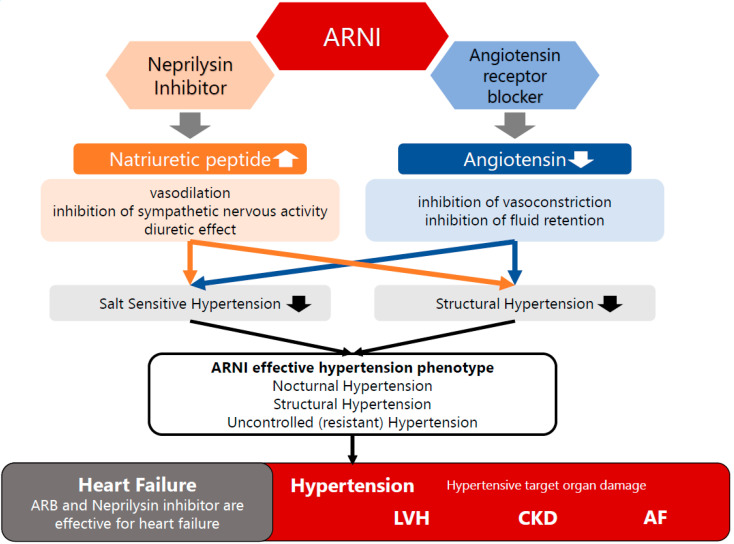
Mechanisms of the efficacy of ARNI for elevated BP and congestive heart failure. Angiotensin receptor neprilysin inhibitor (ARNI) has (1) an inhibitory effect on angiotensin and (2) an inhibitory effect on neprilysin. ARNI has been reported to have a variety of effects such as suppressing excessive sympathetic nerve activity and vasoconstriction and decreasing fluid retention. These multiple effects of ARNI are useful in the pathogenesis of nocturnal hypertension, structural hypertension, and treatment-resistant hypertension.

ARNI was developed primarily for the treatment of heart failure and is applied for the treatment of heart failure in Europe and the U.S. and the treatment of hypertension in Japan. ARNI has (1) an inhibitory effect on angiotensin and (2) an inhibitory effect on neprilysin, which is the enzyme that degrades b-type natriuretic peptide (BNP). Thus, ARNI has been reported to have a variety of effects, such as suppressing excessive sympathetic nerve activity and vasoconstriction and decreasing fluid retention. These multiple effects of ARNI are useful in the pathogenesis of nocturnal hypertension, structural hypertension, and treatment-resistant hypertension (Figure 3). ARNI is useful in hypertensive organ disorders, including hypertensive heart disease [50]. Previous studies have documented the antihypertensive effects of ARNI. In an evaluation using ABPM, ARNI has also been reported to lower BP consistently over a 24 h period, during the daytime as well as the nighttime [51]. Moreover, ARNI (sacubitril/valsartan) has demonstrated superiority over ARB (valsartan) in reducing BP among patients with treatment-resistant hypertension. The reduction in systolic BP at week 16 was greater with sacubitril-valsartan vs. valsartan in patients with treatment-resistant hypertension (3.9 [−6.6 to −1.3] mmHg) and apparent MRA-resistant hypertension (−6.3 [−12.5 to −0.1] mmHg) [52].

### 5.2. Sodium Glucose Cotransporter 2 Inhibitor (SGLT2i)

SGLT2i has been shown to contribute to the prevention of CVD in patients with diabetes and heart failure [54,55,56,57,58]. SGLT2i can be used for two types of disease, such as diabetes and heart failure, via a single pill, which is considered a treatment consistent with the concept of polypill strategy. Furthermore, there is evidence to suggest that SGLT2 inhibitors may have a positive impact on the cardio-renal prognosis of patients with chronic renal failure in the context of hypertension research [59]. It has been reported that empagliflozin had the effect of lowering 24 h BP levels in patients with diabetes and uncontrolled nocturnal hypertension in a randomized controlled trial (after 12 weeks, 24 h ambulatory BP level was −7.7 mmHg in the interventional group versus the placebo group) [53]. In addition, canagliflozin has been reported to have the effect of lowering BP in patients with diabetes and chronic kidney disease [60]. From these study findings, SGLT2i is suggested to be appropriate for use in the treatment of diabetes and heart failure, and for the control of elevated BP [53,60,61]. The therapeutic effect of SGLT2i on many of these diseases may be considered as one possible polypill treatment strategy.

## 6. Problems and Limitations of Polypill Therapy

The advantages of a “one-size-fits-all” treatment strategy such as polypill therapy include (1) improved medication adherence, (2) a certain quality of treatment even in patients with low health literacy, and (3) effectiveness in preventing cardiovascular disease in low-income areas [3,62,63,64]. However, the recommendation of a “one-size-fits-all” treatment strategy for cardiovascular disease prevention, such as the polypill strategy, is inconsistent with the modern direction of precision medicine, which develops individualized treatment strategies based on a combination of clinical characteristics and genomic and lifestyle factors [65]. In the field of hypertension, the usefulness of artificial intelligence and machine learning for the early diagnosis of hypertension and the risk stratification of individual patients has been reported in recent years [66,67,68]. In addition, risk factors for nocturnal hypertension have been reported as a patient-specific pathophysiologic assessment [69]. Treatment management that takes into account the individual patient’s pathophysiology is recommended [66,70]. In addition to traditional drug treatments, new therapies such as renal denervation and digital therapeutic applications for behavioral modification are being developed [68,71,72,73]. It is important to individualize treatment by combining these different treatment modalities, taking into account the response to each treatment. These treatment modalities are the opposite of one-size-fits-all polypill therapy. Clinical practitioners need to be aware of the advantages and disadvantages of polypill therapy.

## 7. Conclusions

Evidence is accumulating on the benefits of polypills in cardiovascular disease prevention. Novel therapeutic agents such as ARNI and SGLT2i with multimodal therapeutic effects may also be an avenue for polypill treatment strategies. Combination tablets are of particular importance in the field of hypertension treatment. Hypertension guidelines emphasize the use of combinations of multiple antihypertensive medications, and thus the use of combination forms is expected to improve medication compliance and provide stronger antihypertensive effects.

## Figures and Tables

**Table 1:** Major clinical trials designed to compare between polypill and usual care (multiple tablets administered separately) for medication adherence and cardiovascular outcome.

Trial	Year	Confirmation of Polypill	Primary or Secondary Prevention	Number of Patients	Findings
CRUCIAL	2011	amlodipine, atorvastatin	-	1461	Lower BP and cholesterol with polypill than usual care (UC). Framingham 10-year CHD risk 13% with polypill vs. 16% in usual care.
UMPIRE	2013	aspirin, simvastatin, lisinopril, atenolol or hydrochlorothiazide	Primary and secondary	2004	Lower BP and cholesterol with polypill than UC. There is no difference in major CVD events at median 15 mo. follow-up: 50 (5%) with polypill vs. 35 (3.5%) in UC, RR 1.45, 95%CI 0.94–2.29, *p* = 0.09 (NS)
IMPACT	2014	aspirin, simvastatin, lisinopril, atenolol or hydrochlorothiazide	Primary and secondary	513	Improved adherence with polypill. No difference in BP and LDL-cholesterol between polypill and UC. There is no difference major CVD events at 12 mo. follow-up: 16 with polypill vs. 18 in UC, *p* = 0.73 (NS)
Kanyini GAP	2014	aspirin, simvastatin, lisinopril, atenolol or hydrochlorothiazide	Primary and secondary	623	Improved adherence with polypill. No difference in BP and LDL-cholesterol between polypill and UC.
FOCUS	2014	aspirin, simvastatin. ramipril	Secondary	2118	Improved adherence with polypill. No difference in BP and LDL-cholesterol between polypill and UC
SPACE	2016	aspirin, simvastatin, lisinopril, atenolol or hydrochlorothiazide	Primary and secondary	3140	Combination of three trials (UMPIRE, Kyayini GAP, and IMPACT) for polypill. Improved adherence with polypill. Lower BP and cholesterol with polypill than UC.
PolyIran	2022	aspirin, atorvastatin, hydrochlorothiazide, enalapril or valsartan	Primary and secondary	6838	Polypill is associated with reduced major CVD events at 60 mo. follow-up: 202 (5.9%) with polypill vs. 301 (8.8%) in UC, HR 0.66, 95%CI 0.55–0.80. Improved adherence with polypill.
SECURE	2022	aspirin, ramipril, atorvastatin	Secondary	2499	Polypill is associated with reduced major CVD events at 36 mo. follow-up: 118 (9.5%) with polypill vs. 156 (12.7%) in UC, HR 0.76, 95%CI 0.60–0.96, *p* = 0.02.

CRUCIAL, Cluster Randomized Usual Care vs. Caduet Investigation Assessing Long-Term-Risk; FOCUS, Fixed-Dose Combination Drug for Secondary Cardiovascular Prevention; IMPACT, IMProving Adherence using Combination Therapy; Kanyini GAP, Kanyini Guidelines Adherence to Polypill; SECURE, Secondary Prevention of Cardiovascular Disease in Elderly; SPACE, Single Pill to Avert Cardiovascular Events; UMPIRE, Use of a Multidrug Pill in Reducing Cardiovascular Events.

## Data Availability

As this is a review article, there is no data available on this paper.
